# Exosome-derived microRNAs in cancer metabolism: possible implications in cancer diagnostics and therapy

**DOI:** 10.1038/emm.2016.153

**Published:** 2017-01-20

**Authors:** Marco Tomasetti, Wan Lee, Lory Santarelli, Jiri Neuzil

**Affiliations:** 1 Department of Clinical and Molecular Sciences, Polytechnic University of Marche, Ancona, Italy; 2 Department of Biochemistry, Dongguk University College of Medicine, Gyeongju, Korea; 3 Mitochondria, Apoptosis and Cancer Research Group, School of Medical Science and Menzies Health Institute Queensland, Griffith University, Southport, Queensland, Australia; 4 Molecular Therapy Group, Institute of Biotechnology, Czech Academy of Sciences, Prague-West, Czech Republic

## Abstract

Malignant progression is greatly affected by dynamic cross-talk between stromal and cancer cells. Exosomes are secreted nanovesicles that have key roles in cell–cell communication by transferring nucleic acids and proteins to target cells and tissues. Recently, MicroRNAs (miRs) and their delivery in exosomes have been implicated in physiological and pathological processes. Tumor-delivered miRs, interacting with stromal cells in the tumor microenvironment, modulate tumor progression, angiogenesis, metastasis and immune escape. Altered cell metabolism is one of the hallmarks of cancer. A number of different types of tumor rely on mitochondrial metabolism by triggering adaptive mechanisms to optimize their oxidative phosphorylation in relation to their substrate supply and energy demands. Exogenous exosomes can induce metabolic reprogramming by restoring the respiration of cancer cells and supress tumor growth. The exosomal miRs involved in the modulation of cancer metabolism may be potentially utilized for better diagnostics and therapy.

## Introduction

Solid tumors are composed of cancer cells surrounded by extracellular matrix (ECM) that supports the tumor vasculature and a wide range of host-derived cells, including cancer-associated fibroblasts (CAF), lymphocytes and myeloid cells that coexist in a dynamic and adaptive environment. Activated CAFs synthesize, deposit and alter the three-dimensional ECM scaffold by secreting collagens and matrix-modifying enzymes, facilitating cancer cell proliferation and metastasis via paracrine growth factors and chemokines.^1^ Adaptive communication is particularly important between cancer cells and the local and distant environment. Recently, extracellular vesicles (EVs) have emerged as long-distance communicators; their effects in primary tumors can also have systemic effects and contribute to processes within the circulation.^2^ Exosomes represent a special class of EVs, which are released by a variety of cells.^3, 4^ Cancer cells produce higher amount of exosomes with respect to their non-malignant counterparts.^5^ Tumor-released exosomes induce alterations in their recipient cells, thereby playing a role in tumor growth, angiogenesis and metastasis.^6^ The mechanism by which exosomal cargo is selected is not known. However, determinant factors for exosomal content are the type of the donor or recipient cell, as well their state. Exosomes have been shown to transport proteins, lipids and nucleic acids (DNA, mRNA, miRs). Increasing evidence has implicated exosome-delivered miRs in cancer cell communication, which is an important and complex process allowing tumor cells to ‘shape' and influence their environment. This review will focus on the role of exosome-containing miRs in metabolic re-programming associated with cancer and their involvement in the complex interplay between cellular and non-cellular components of the tumor stroma.

### Tumor microenvironment and cancer metabolism

Tumors are very complex tissues composed of heterogeneous subpopulations of cancer cells associated with stromal components such as fibroblasts, mesenchymal cells (MSCs), smooth muscle cells, pericytes, (myo)-fibroblasts, immune cells, platelets and endothelial cells (ECs). Cancer cells, by means of secretion of soluble factors, cytokines and exosomes, continuously remodel their environment by the recruitment and activation of surrounding cells.

Fibroblasts in tumors are key players in the process of tumorigenesis.^7^ It was reported that normal quiescent fibroblasts inhibit the growth of neoplastic cells via direct contact or by their ability to maintain epithelial homeostasis and proliferative quiescence.^8, 9^ Hypoxia, reactive oxygen species (ROS), as well as oncogenic signaling within tumors frequently drive the recruitment of normal-associated fibroblasts (NAFs), reprogramming them into cancer-associated fibroblasts (CAFs).^10^ CAFs constitute approximately one-third of the stromal mass to form a continuous ‘sheet' surrounding tumor blood vessels. Several tissues contribute to the population of CAFs.^11^

The most direct source of CAFs is resident tissue fibroblasts and MSCs.^12^ Other potential sources of CAFs are the stellate cells and ECs undergoing the process of endothelial–mesenchymal transition.^13^ Functionally, CAFs promote tumor growth through a paracrine mechanism by their production of a wide variety of ECM molecules and cytokines.^14^ CAFs secrete multiple soluble factors, such as vascular endothelial growth factor A (VEGF-A), hepatocyte growth factor (HGF), epidermal growth factor (EGF), platelet-derived growth factor (PDGF), nerve growth factor, insulin-like factor (IGF), basic fibroblast growth factor (bFGF or FGF2) and members of the Wnt family (Chaffer and Weinberg).^10, 15^

Cytokines and VEGF attract monocytes and bone marrow-derived cells into the tumor environment, which differentiate into myofibroblasts/fibroblasts and tumor-associated macrophages, respectively.^16^ A subset of bone marrow-derived cells express α-smooth muscle actin (α-SMA), indicating that bone marrow-derived cells are activated and involved in tissue repair.^13^ The induction of α-SMA alters cytoskeletal organization, which increases the contractile ability of myo-fibroblasts. Tumor-associated macrophages are the major cellular component of cancer-related inflammatory reactions, having served as a paradigm for the plasticity and functional polarization of mononuclear phagocytes. Tumor-associated macrophage cells can secrete different matrix metalloproteinases (MMPs), which can degrade the ECM, leading to the release of additional matrix-sequestered growth factors and providing activated ECs with space to migrate to.^17^ Similarly, differentiated tumor-associated neutrophils, contribute to ECM remodeling by the secretion of MMP9, which leads to the release of matrix-bound growth factors such as VEGF.^18^ Functionally, MMPs have been linked to tumor angiogenesis and metastasis.^19^

The tumor vasculature is described as chaotic and torturous, irregular in the lumen diameter, dilated and highly permeable, deficient in pericyte coverage and abnormal in endothelial lining. Studies revealed that tumor endothelial cells are different from normal endothelial cells from the molecular and functional point of view.^20^ Compared with the normal endothelial cells, tumor endothelial cells were shown to be more responsive to angiogenic factors such as FGF2 and VEGF.^21^ Furthermore, high levels of EGFR, which is not expressed in normal endothelial cells, were found in tumor endothelial cells.^22^ Recent evidence indicates that cells of the vascular endothelium are heterogeneous and exhibit specialized phenotypes depending on their organs of origin and functional state.^23, 24^ Cytogenetic abnormalities can occur through horizontal transfer of genomic material between ECs and tumor cells.^25^ Many transcriptional changes occur in stromal cells, including epigenetic changes affecting gene and miR expression, thereby inducing a shift in the metabolome and secretome.^26, 27^

Stromal and cancer cells undergo a reciprocal metabolic reprogramming useful to sustain cancer cells survival and growth. Recently, a metabolic shift from oxidative phosphorylation toward glycolysis in cancer cells and from glycolysis toward oxidative phosphorylation in fibroblasts was found in the co-culture of human cervical carcinoma cells and human fibroblasts. The metabolic switch was accompanied by hydrogen peroxide production and slight acidification of the cytosol in the cancer cells in comparison with that of the corresponding monoculture.^28^ It has been reported that CAFs actively participate in the complex metabolism of tumors by engaging a biunivocal relationship with cancer cells forcing them to respire and overcome energy depletion by means of the Warburg effect.^29^ In this context, mitochondrial reprogramming has a mandatory role in cancer cells, leading to a shift toward ketone body/glutamine utilization and citrate-mediated fatty acids synthesis. Downregulation of isocitrate dehydrogenase 3 (IDH3a) decreased the level of α-ketoglutaric acid (α-KG) by reducing the ratio α-KG to fumarate and succinate, resulting in the stabilization of prolyl hydroxylase domain-containing protein-2 (PHD2) and the hypoxia-inducible factor 1-α (HIF1α). This behavior commits stromal cells to a less-efficient metabolism, and cancer cells to exploit stromal cells to sustain survival and growth in hypoxic and hypo-nutrient conditions.

**Figure 1:**
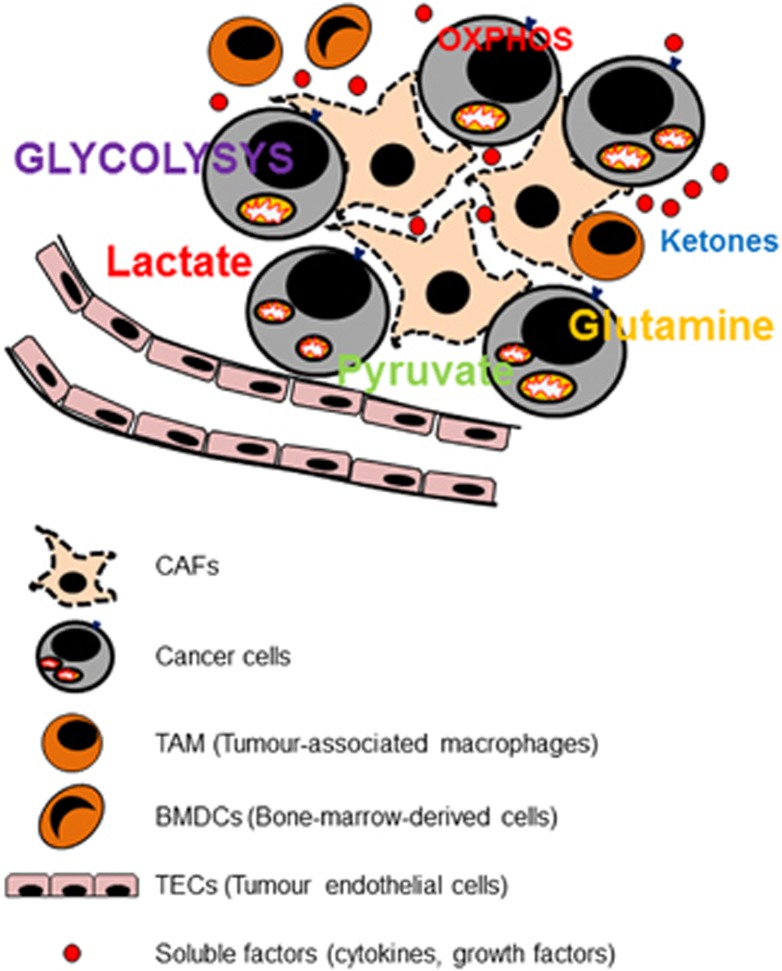
Metabolic symbiosis between cancer cells and cellular components of stroma: cancer-associated fibroblasts (CAFs), tumor-associated macrophages (TAM), bone-marrow-derived cells (BMDCs) and tumor endothelial cells (TECs). In the tumor microenvironment, cancer cells are prone to glycolysis and the adjacent CAFs adapt to glycolysis. Metabolic intermediates such as lactate, pyruvate, ketones and glutamine secreted by CAFs can be used by cancer cells for the biosynthesis of macromolecules. The acid environment generated activates matrix metalloproteinase (MMPs) and prevents immune attack, thus providing cell growth and metastatic dissemination.

Cancer cells create ‘pseudo-hypoxic' conditions for fibroblasts by inducing HIF1α, thus promoting glycolysis. Metabolic symbiosis between cancer cells and CAFs requires that the cells express a subtype of monocarboxylate transporter 1 (MCT1), which contributes to the uptake of lactate provided by CAFs expressing MCT4.^30^ Cancer shows a high metabolic heterogeneity; MCT4-expressing tumor cells produce and secrete lactate from glycolysis, while MCT1-expressing cancer cells import lactate via MCT1 and perform oxidative phosphorylation.^31^ Cancer cells synthesize pyruvate from lactate, providing the tricarboxylic acid cycle with intermediate metabolites. Therefore, a subpopulation of cancer cells that depend on aerobic glycolysis takes up and use glucose at high rates, whereas another subpopulation engages in oxidative phosphorylation and glutaminolysis by means of activated mitochondrial metabolism. The metabolic relationship among the cellular components of stroma is summarized in Figure 1.

Glutaminolysis represents a series of biochemical reactions by which glutamine is catabolized into downstream metabolites, such as α-KG and glutamate. α-KG enters the tricarboxylic acid cycle and is catabolized to malate, which is transported into the cytoplasm, converted to pyruvate and then to lactate.^32^ SIRT4, a mitochondria-localized member of the sirtuin family, is a critical negative regulator of glutamine metabolism.^33^ Mechanistically, mTOR-signaling pathway represses SIRT4 by promoting the proteasome-mediated destabilization of cAMP-responsive element binding-2. Both aerobic glycolysis and glutaminolysis are simultaneously activated in malignant cancer cells.^34^ Recently, it has been reported that a MYC transcriptional target mediates elevation of glutaminolysis, being essential for lactate transport and glycolytic flux in several cancer cell lines.^35, 36^ Lactate in the extracellular space promotes the acidic condition, which in turn leads to pseudo-hypoxia. Under hypoxia PDGF-BB is highly upregulated, leading to the activation of the phosphatidylinositol 3-kinase (PI3K)/Akt pathway.^37^ AKT activation occurs at a higher rate and has been described in a variety of cancer types including malignant mesothelioma.^38, 39^

### Tumor-derived exosomes in cell-to-cell communication

The cross-talk between the stromal populations is not only mediated by soluble cytokines and growth factors, but is also promoted by metabolites, such as lactate, ketone bodies or proteins. These metabolites are exchanged among stromal and cancer cells by means of membrane solute transporters, or mediated by the release of vesicles such as exosomes. Normal and cancer cells may release membrane-bound nanovesicles into the extracellular space and body fluids. These membrane-derived vesicles (extracellular vesicles, EVs) can be divided into three main classes depending on their sizes, that is, exosomes (20–100 nm), microvesicles (100–1000 nm) and apoptotic bodies (1–5 μm). Differently from the other cellular vesicles such as apoptotic bodies that bud off the plasma membrane, exosomes are of endocytic origin. Exosomes are formed as intraluminal vesicles by a process that involves the endosomal system and are secreted upon fusion of endosomal multivesicular bodies (MVBs) with the plasma membrane.^40, 41^ The conversion of the intraluminal vesicles into MVBs is mediated by the endosomal sorting complex required for transport, which involves the lateral segregation of cargo at the endosomal limiting membrane, the formation of an inward budding vesicle and the release in the endosomal lumen of the membrane vesicle containing a small portion of cytosol.^42^

Although the endosomal sorting complex required for transport system is generally thought to be the main pathway of exosomal biogenesis, several studies have shown the existence of endosomal sorting complex required for transport-independent biogenesis of exosomes. Other mechanisms of exosome biogenesis may operate in parallel to the endosomal sorting complex required for transport machinery, which is based on the cell type and the specific lipid composition of the endosomal membrane.^42^ Once formed, MVBs are either destined for degradation and secretion, both being governed by Rad GTPases. It was reported that Rad7 could mediate the degradation via MVBs and lysosomal fusion, while other Rab proteins are involved in intracellular trafficking and secretory events.^43^ The final release of intraluminal vesicles occurs upon MBVs fusion with the plasma membrane by involving the soluble *N*-ethylmaleimide-sensitive factor attachment protein receptors.^44^ The release of exosomes may be facilitated by membrane invagination of intraluminal vesicles promoted by certain lipids. In this context, the inhibition of neutral sphingomyelinase (nSMase), a protein responsible for the production of ceramide, could reduce the release of exosomes. However, in certain cell types, the depletion of nSMase does not inhibit the formation of MVB or exosome.^45^

The capacity to secrete exosomes differs for individual cell types and can be constitutive or inducible. Cells that constitutively secrete exosomes are dendritic cells and macrophages, while mast cells and T cells need to be activated.^41^ In cancer cells, genotoxic stress induces exosomal secretion through a mechanism that involves p53 activation and increased product of the tumor suppression-activated pathway 6 (TSAP6) genes.^46^ During the biogenesis of exosomes and before their secretion, various molecules are uploaded into the lumen. These molecules include proteins of the major histocompatibility complex class I and II, proteases, cytokines, growth factors as well as death ligands. Exosomes can also contain genetic information such as DNA, mRNA and regulatory miRs. Their molecule profile can be divided into two groups: proteins relevant for the biogenesis and secretion of exosomes, and molecules that are specifically uploaded into vesicles by certain cell types that provide exosomes with a characteristic cell type-specific ‘fingerprint'.^47^ Specific sorting of proteins and RNA molecules into exosomes is controlled by a variety of pathways, most of which are not fully understood. It has been clarified that the composition of exosomes will determine the outcome of the associated intercellular communication. The ubiquitination process and the plasma membrane anchor tags provided by myristoylation, prenylation or palmitoylation have a role in protein shuttling into exosomes.^48^ In addition, CD43, a transmembrane glycoprotein, has been implicated in the selective protein upload into exosomes; CD43 interacts with DICER, which is uploaded into EVs.^49^

RNAs are not randomly loaded into exosomes. MiRs can be uploaded into exosomes based on specific ‘shuttle' sequences. SUMOylated heterogeneous nuclear ribonucleoprotein A2B1 (hnRNPA2B1) specificity binds to miRs containing the ‘shuttling' motif GGAG, leading to their upload into exosomes.^50^ MiRs are enriched in exosome under high level of expression of individual miRs and low level of expression of their cognate target mRNA.^51^ In addition, AGO2, a protein associated with the RNA-induced silenced complex (RISC) complex, is thought to control the loading of miRs into exosomes.^52^ Exosomes can thus act as mediators of cell-to-cell communications via direct exchange of genetic material between cells.^47^ Exosome-mediated communication is very important for tumor cells, which constitutively secrete exosomes that have an important role in the modulation of the immune response against tumors,^53^ induction of angiogenesis,^54^ and cell invasion and metastasis.^55^ Tumor cells are continuously subjected to a range of stressors such as hypoxia, starvation or chemotherapeutic agents, and cancer progression depends on the ability of cells to sense and adapt to these situations. Immune cells release miR-containing exosomes that can be then taken up by recipient cells.

Cells internalize exosomes either by fusion with the plasma membrane or via endocytosis.^56^ Uptake of exosome is mediated via mechanisms involving protein interactions that facilitate subsequent endocytosis.^57, 58^ Binding of exosomes to the surface of recipient cells is mediated by the classical adhesion molecules involved in cell–cell interactions, such as integrins and ICAMs. However, other more specific proteins and membraneous structures have a role, such as tetraspanin-enriched microdomains that are clusters of tetraspanins, adhesion molecules and cognate transmembrane receptor proteins, located in raft-like structures at the plasma membrane.^59^ The heparin sulphate proteoglycans on the cell surface are important for mediating vesicular entry.^57^ T-cell immunoglobulin mucin-binding phosphatidylserines, carbohydrate/lectin receptors and heparin sulphate proteoglycans could be involved as well. ICAM1–LFA1 interactions are involved in exosome uptake by immune cells.^60^ Most of experimental evidence suggests that exosomes are usually taken up into endosomal compartments via endocytosis. The process of endocytosis includes clathrin-dependent endocytosis and clathrin-independent pathways, such as caveolin-mediated uptake, macropinocytosis, phagocytosis and lipid raft-mediated internalization.^56^ Internalization of exosomes is not a passive process; their uptake is energy-dependent and requires functional cytoskeleton.^61^

Several studies show that fluorescently labeled EVs can be taken up by virtually any cell type,^58^ whereas others suggest that vesicular uptake is a highly specific process, which can only occur if the cell and the EV share the right combination of a ligand and a receptor. However, multiple mechanisms are responsible for exosome-cell communications, and different communication strategies are used by individual cell type. Endocytosis, representing the most important mechanism of endosomal uptake, is regulated by different pathways including PI3K signaling. The inhibition of PI3K by wortmannin markedly reduced uptake of exosomes.^62^

### Exosome-delivered miR in orchestrating tumor microenvironment metabolism

**Figure 2:**
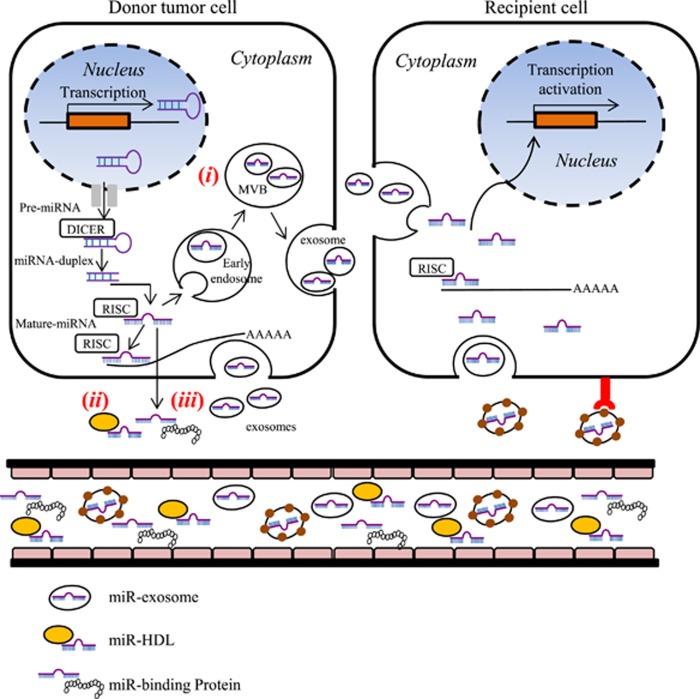
miRNA biogenesis and release mechanism to recipient cells. miRNA genes are transcribed in the nucleus in pre-miRNAs and exported into cytoplasm. The DICER complex by cleavage generates an intermediary miRNA duplex, of which one strand is incorporated into RNA-induced silencing complex (RISC) to form mature miRNA. A fraction of miRNAs are released from cells into the extracellular environment (i) within multi-vesicular bodies (MVB) and secreted via exosomes; (ii) incorporated into high-density lipoproteins (HDL) particles; (iii) associated with RNA-binding proteins, such as AGO2 and released of the miRNA–AGO complex. Exosome miRNAs (exo-miRs) are involved in cell-to-cell communication. Exo-miRs are released into the extracellular compartment acting as autocrine/paracrine mechanism, or in the blood stream with endocrine effect. Exosome containing miRNAs are uptaken and internalized into cytoplasm of recipient cells through endocytosis pathway. The exosome uptake mechanisms involve protein interactions that facilitate subsequent endocytosis.

In physiological and pathological conditions, exosomes act as multi-molecular messengers. The mechanism of exosome-mediated cell–cell communications is particularly important in cancer, as tumor cells constitutively secrete exosomes that can target adjacent cells of the same type (autocrine effect), neighboring cells of different types (paracrine effect) or reach cells located at distant organs after entering the bloodstream (endocrine effect). The main functions of exosomes in the cancer microenvironment include the following: promotion of primary cancer growth, stimulation of angiogenesis, activation of stromal fibroblasts, sculpting the cancer ECM, generation of a pre-metastatic niche and suppression of the host immune response. In this context, miR-based intercellular communication relies on several critical processes. Exosomes protect their cargo from enzymatic degradation during transit through extracellular environment.^63^ Upon release of their functionally active miR load inside the recipient cell, exosomal cargo can regulate gene expression via *de novo* translation and post translation regulation of target mRNAs (Figure 2).^2, 64^

Several studies confirmed the presence of horizontal transfer of miR via exosomes derived from glioblastoma,^65^ lung cells,^66^ endothelial cells^67^ and MSCs.^68^ More specifically, lung cancer-derived exosomes were found to be enriched in mRNA, miR and pro-inflammatory proteins and, following horizontal transfer of these molecules, promoted inflammatory phenotype in growing tumors and stimulated tumor cell proliferation.^69, 70^ MiRs transferred by exosomes are emerging as novel regulators of cellular function including cell metabolism.

Recently, the miR-126 that is known to regulate angiogenesis has been found to control cancer metabolism by targeting the insulin receptor substrate-1 (IRS1).^39, 71^ IRS1 is an adaptor proteins involved in signaling via insulin receptor (IR) and insulin-like growth factor I receptor (IGF-IR). In addition to its metabolic and growth-promoting functions, IRS1 has a role in malignant transformation. Alterations in IRS expression have been documented for certain neoplastic diseases, such as malignant mesothelioma, and hepatocellular, pancreatic and breast cancer.^39, 72, 73, 74, 75^ Although the mechanism by which IRS-1 supports tumor growth is not fully understood, a plausible hypothesis is that IRS-1 amplifies the signal of the IGF-1R. The IGF axis is a complex signaling network that is involved in many physiological and pathological processes such as mitogenesis, angiogenesis, transformation, differentiation, tissue homeostasis, and regulation of apoptosis and cell motility.^76^ For instance, steroids, cytokines, hormones and integrins all have been shown to regulate IRS function.^77^ Expression of the IRS protein can be regulated by different miRs in response to both mitogenic and metabolic.^78, 79, 80, 81^ MiR-126 is highly expressed in ECs, and has been shown to be secreted into the tumor surrounding milieu.^82, 83^

Exosomal transfer of miR-126 has functional relevance. ECs release exosomes enriched in miR-126, which are taken up by ECs (paracrine signaling), or translocate to other compartments to modulate the downstream intercellular signaling mediators. Notably, high glucose treatment or diabetes reduces miR-126 levels in peripheral blood mononuclear cells, and this is associated with impaired pro-angiogenic properties.^84^ On the other hand, oxidative stress and glucose deprivation increase miR-126 encapsulated in exosomes.^39, 85, 86^ Downregulation of miR-126 was found to inversely correlate with increased microvessel density and VEGF-A expression in gastric cancer tissues.^87^ Increasing evidence suggests that miR-126 participates in glucose homeostasis via its targets.^88^ Ectopic miR-126 reduced mitochondrial respiration and promoted glycolysis, reducing Akt signaling, inhibiting cytosolic sequestration of FoxO1, and promoting the expression of genes involved in gluconeogenesis and oxidative stress defense in malignant mesothelioma cells. Cells expressing miR-126 feature high level of mitochondrial SOD2 and CAT, also regulated by FoxO1.^39, 71, 89^ Enhanced ROS production in cancer drives the onset of aerobic glycolysis, with lactate and ketone production promoting mitochondrial biogenesis and anabolic growth of tumor cells. Alleviation of mitochondrial oxidative stress via enhanced expression of antioxidant enzymes targeted to mitochondria was found to be sufficient to lower tumor severity and to considerably reduce the tumor burden, linking miR-126 to the suppression of the onset and progression of cancer.

Akt activates ATP citrate lyase (ACL), promoting the conversion of mitochondria-derived citrate to acetyl-CoA for lipid synthesis.^90^ ACL links glucose to lipid metabolism, being responsible for conversion of citrate to cytosolic AcCoA, an important component of several biosynthetic pathways. AcCoA is the substrate for *de novo* synthesis of lipids and for protein acetylation. Ectopic expression of miR-126 resulted in low citrate levels by inhibiting ACL. This mechanism favors glucose oxidation to produce energy rather than converting it into precursors for biosynthetic pathways. Restored citrate, induced by ACL inhibition, is linked to HIF1α activation and stabilization.^39, 81^ Several miRs that mediate metabolic re-programming can contribute to HIF-1α expression and stabilization.^91^ In chronic lymphocytic leukemia, stabilization of HIF-1α under normoxia is mediated by miR-92-1, which targets the VHL tumor suppressor,^92^ an E3 ubiquitin ligase involved in the degradation of HIF-1α in the presence of oxygen. Under decreased oxygen availability, miR-424 upregulation in ECs stabilizes HIF-1α via targeting cullin-2, a scaffold protein critical for the assembly of the ubiquitin ligase system.^93^

In cancer cells, majority of AcCoA is derived from pyruvate via PDH.^94^ Therefore, mitochondrial activity involving the pyruvate–citrate shuttle is a critical step for the biosynthesis of fatty acids (FAs) and cholesterol, and for protein acetylation. PDH flux is regulated by cyclic phosphorylation and dephosphorylation of specific PDKs and pyruvate dehydrogenase phosphatases (PDPs), whose function is regulated by cellular nutrients. MiR-126 was found to reduce PDK expression while, paradoxically, inhibiting PDH activity, which increased the level of pyruvate in the cytosol.^71^ Under these conditions, total glucose oxidation via the tricarboxylic acid cycle is rather low, and the energy demand is primarily met by FA and ketone body oxidation.

Reprogrammed glucose metabolism as a result of increased glycolysis and glucose uptake is a hallmark of cancer. Cancer cells can suppress glucose uptake by non-tumor cells in the pre-metastatic niche by secreting vesicles that carry high levels of the miR-122.^95^ Hence, miR-122, taken up by surrounding cells, targets PKM2 and represses glycolytic metabolism, thereby lowering glucose utilization by the niche cells and allowing glucose use by growing cancer cells.^95^ Adaptation of a pre-metastatic niche prior to the ‘arrival' of tumor cells has been recognized as an important means for cancer to facilitate its sustained progression and the ensuing metastasis.^96^ For instance, alteration of glucose consumption by the niche cells could lead to reprogramming energy demands to induce cancer progression via cancer-derived extracellular miR-122. This is an example of nutrient utilization in the context of cancer-host crosstalk. Cancer cells systemically suppress nutrient utilization by other cell types to gain advantage. This miR-122-mediated process may be more important at an early stage of tumor formation then in later stages characterized by high rate of angiogenesis, due to the limited availability of nutrients in the tumor microenvironment that cannot sustain tumor growth, and when disseminated tumor cells arrive to distal sites in order to rapidly expand.

Recently, it has been shown that tumor suppressor miRs, released from normal prostrate cells, can transfer growth inhibitory signals to prostate cancer cells.^97^ Normal cells secrete anti-proliferative miRs in an attempt to maintain normal miR homeostasis. However, the aberrant cancer cells circumvent this inhibitory effect resulting in the expansion of the tumor. In this context, miR-122 transfers from hepatic cells expressing it at high levels to HepG2 cells with reduced levels of miR-122. HepG2 cells overcome the effect exerted of imported miR-122 by secreting IGF1, which in turn inhibits miR-122 biogenesis in neighboring cells. This response to high level of miR-122 produced by neighboring cells may be a strategy adapted by liver cancer cells to modulate their microenvironment toward their benefit, resulting in better proliferation. Exosomal delivery of miR-122 to hepatoma cells may serve as a mechanism for maintaining miR homeostasis. In addition, miR-122 in secreted exosomes can mediate communication between adipose tissue and hepatocellular carcinoma.^98^ miR-122 acts as an important regulator of cholesterol and FA metabolism.^99^ This miR has been also described to stimulate the production of endoplasmic reticulum (ER)-associated lipid droplets and formation of cholesterol-rich membrane domains; inhibition of miR-122 may contribute to a shift in the equilibrium between lipid storage and metabolism.^100^

Cancer-derived exosomes from cells or serum of patients were found to contain the RISC-loading complex proteins, DICER, TRBP and AGO2, which are involved in miR biosynthesis and promote tumorigenesis.^49^

### Exosome-derived miRs as circulating biomarkers in cancer

Exosomal miR (exo-miR) profiling of serum from cancer patients versus healthy individuals has revealed important differences in relation to tumor progression, highlighting a possible use of these miRs as disease prognostic biomarkers.^101^ Exosomes are a stable source of miR in bodily fluids, preventing degradation of biological macromolecules under non-physiological conditions.^102^ In fact, exosomally derived miR has been found to remain stable at −208 °C for 5 years and to be resistant to freeze-thaw cycles.^103^ This high stability implies that miRs can be used for cancer screening or as non-invasive biomarkers for monitoring of the disease.^104^ However, detection of exo-miRs experiences similar problems as those encountered in the case of more conventional tumor biomarkers; for example, the exosomes secreted by other cell types can potentially mask their cancer-specific counterparts. In fact, cancer cell-derived exosomes differ greatly in their level and functional effects compared with exosomes derived from non-cancerous cells.^49^

Despite a number of methods for extraction of exosomes and quantification of miR, their applicability for diagnostics in a clinical setting is questionable. For effective biomarker analysis of exosomes, pure samples are required. An additional limitation to the use of exo-miRs as biomarkers is the high variation in exo-miR levels due to the wide ranging cycle threshold values obtained by qRT-PCR.^105, 106^

MiRs, detected in exosomes from serum of patients with breast cancer, can distinguish specific molecular subtypes.^105^ This study indicated that higher exosomal levels of miR-373 in breast cancer are indicative of the triple-negative type of the disease, highlighting the potential role of the serum-specific exo-miR-373 as a biomarker for aggressive neoplasias. Identification of exo-miRs associated with distinct metastases could provide an additional diagnostic tool to evaluate the disease stage and monitor its progression (acting as a prognostic marker). Higher miR-105 levels were found in serum-derived exosomes of breast cancer patients who later developed the metastatic disease.^106^ Similarly, upregulation of miR-210 and downregulation of miR-19a and miR-29c has been observed in exosomes derived from brain metastatic breast cancer and from melanoma.^107^

Serum-derived exo-miR-21 and exo-miR-155 were found to be significantly upregulated in recurrent lung cancer compared to primary cancer. These miRs were also upregulated in serum exosomes of recurrent tumor-bearing animals versus primary tumor-bearing or control animals.^108^ Exosome-encapsulated miR-21 was significantly increased in patients with esophageal squamous cell carcinoma and correlated with advanced tumor classification, positive lymph node status and metastasis.^109^ A recent meta-analysis, including 10 types of cancer, indicated that exo-miR-21 could be considered a general biomarker for cancer.^110^ The diagnostic performance of exo-miR-21 was much better than that of circulating miR-21 in several types of cancer. In spite of this, exo-miR-21 was found not inappropriate for the use in diagnosis of certain cancers, such as hepatocellular cancer, since it did not correlate with stage I and II of hepatocellular cancer.^111^

**Figure 3:**
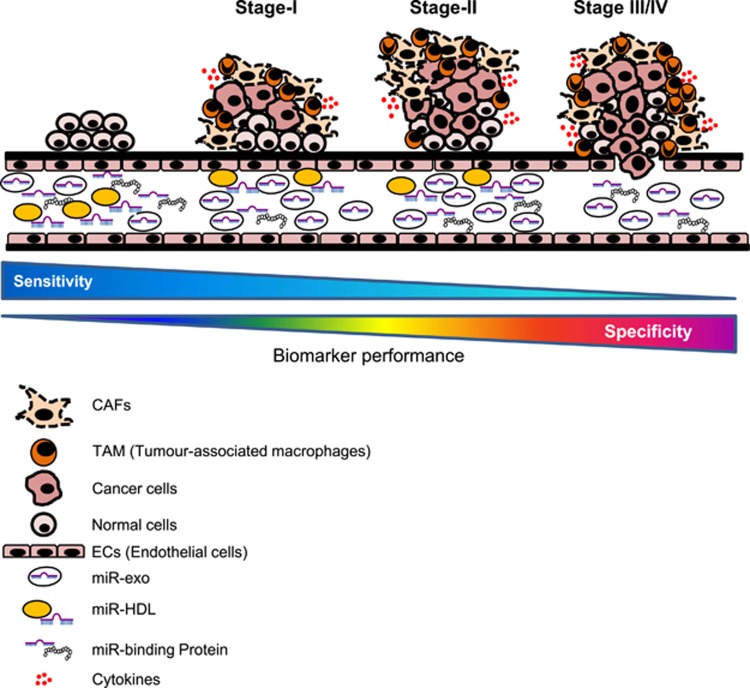
Exosome-miRNAs (Exo-miRs) release from tissue undergoing malignant transformation and tumor progression. During cancer progression, cells release exosome-delivering miRNAs into the bloodstream that can be detected as circulating biomarkers for early detection and progression of cancer.

It has been reported that exo-miR ‘signature' emulates pathological changes in colon,^112^ and prostate cancer.^113^ The exo-miR signature parallels miR expression profiles of the originating tumor cells, indicating that miR profiling can be performed in the absence of biopsy and may rather accurately reflect the tumor's profile.^114^ Preliminary results show that the angio-miR-126, highly expressed in highly vascularized tissues, was found increased in exosomes of early stage non-small cell lung cancer (NSCLC) patients compared with controls and advanced NSCLC subjects. The exosomal transfer of miR-126 to ECs modulates migration and tube formation.^115^ ECs of mature blood vessels express high levels of miR-126, which primarily targets the PI3K regulatory subunit 2 (p85β). As the tumor progresses, the vascular density decreases and tends to be tortuous, unevenly distributed and disorganized. Advanced NSCLC showed low exo-miR-126, resulting in vascular remodeling and EC maturation defects. These data demonstrate that serum exosomes are more informative than the whole serum for evaluating circulating miR-126 levels in patients with NSCLC. Similar results were observed for miR-141 in prostate cancer (PC): exosomal miR-141 better discriminated metastatic PC patients than those with localized PC.^116^ The potential applications of exosome-delivered miRs are various: they can be used for early diagnosis, subtype specification, as well as for the prediction and monitoring the therapy. It has been demonstrated that specific miRs, such as miR-221/222, have a role in resistance to cancer, such as tamoxifen resistance in breast cancer.^117^ Another plausible example is exo-miR-24-3p that is involved in pathogenesis of nasopharyngeal carcinoma and represents a prognostic biomarker for nasopharyngeal carcinoma.^118^ The sensitivity and specificity of exo-miRs according to tumor progression is represented in Figure 3.

### Exosome-mediated miR delivery for cancer therapy

MiRs are key regulators of gene expression, and exo-miRs appear to have a dual role in cancer. On one hand, exosomal delivery of miRs can alter the behavior of the recipient cancer or stromal cells inducing cancer progression and metastasis. On the other hand, exosomes affect molecular pathways that limit the expression of tumor suppressors, facilitating tumor initiation. Therefore, targeting the exosome biogenesis and loading may represent a strategy to treat cancers. The use of amiloride to reduce exosome production and reduce tumor progression was observed *in vivo* for myeloid-derived suppressor cells that inhibit T-cell activation.^53^ This effect was not observed in prostate cancer cells,^119^ suggesting that this mode of inhibition is cell-type-dependent. Another possible mechanism for inhibiting the tumorigenic function of cancer exosomes is to prevent the fusion or uptake of exosomes by target cells. One report documented that tumor-derived exosome uptake by cells can be blocked by diannexin.^120^ However, the low specificity and the possibility to interfere with physiological function of exosomes limit its use. Conversely, the use of exosome as carrier to deliver miRs could become a novel therapeutic approach for cancer.

Exosomes containing miRs represent a promising new therapeutic approach because of their important natural role in cellular processes combined with high stability, tissue-specific expression and secretion into body fluids.^121^ The half-life of exosomes in the circulation is greater than that of liposomes due to their endogenous origin and unique surface composition.^122^ This enables them to specifically bind to recipient cell receptors, providing the possibility to generate exosomes that specifically target a relevant cell type. Moreover, exosomes can carry a variety of cargo, are non-immunogenic, and maintain the cargo stable for delivery.^121^ This notion relates to the development of personalized medicine. Exosomes from MSCs have been used as a vehicle for delivery of anti-tumor miRs. Intra-tumoral injection of exosomes derived from miR-146-expressing MSCs significantly reduced glioma xenograft growth in a rat model of primary brain tumor.^123^ Further, these exosomes inhibit miR-9, resulting in decreased expression of the multidrug transporter that enhances resistance of glioblastoma multiforme cells, sensitizing them to temozolomide.^124^ Another report showed that exosome-derived miR-302b significantly suppressed lung cancer cell proliferation and migration via the TGFβRII/ERK pathway, which is indicative of a novel therapy of lung cancer.^125^

It is also plausible to generate exosomes with therapeutic cargo and ideal surface moieties using semi-synthetic processes to target cell specificity to bypass normal clearance mechanisms. The potential of exosomes as drug delivery carriers can be improved by adding appropriate targeting molecules that can cause accumulation of exosomes at the ‘diseased sites'. For instance, donor cells engineered to express the transmembrane domain of the platelet-derived growth factor receptor fused with the GE11 peptide were used to show that exosomes can be used to efficiently deliver anti-tumor miR to cancer tissues *in vivo*. Intravenously injected exosomes delivered let-7a to EGFR-expressing breast cancer tissue in RAG2^−/−^ mice.^126^ Delivery of anti-miRs by exosomes is a promising strategy, but the pathways by which miRs exert their function must be well characterized to avoid the risk of off-target effects. Exosome-delivered miRs hold a substantial promise to present efficacious personalized therapeutic modalities given their use for biomarker discovery and personalized diagnostics. However, to use exosomes clinically, further studies are needed to resolve a number of contentious issues.

## Conclusions

Exosomes mediate communication between both neighboring and distant cells, thereby emerging as a novel form of intercellular communication, as well as a delivery vehicle. Exosome-shuttled molecules maintain their biological activity, being capable of modulating and reprogramming the recipient cells. For instance, exosomes represent the major delivery system for miRs involved in the communication mechanism between tumor-associated cells and cancer cells. The role of exosome as a novel drug delivery system appears to have several advantages over the existing approaches because of their small size, lack of toxicity and target specificity. Choosing the correct cell line for therapeutic exosome production is of great importance. Proper cell choice can also dictate the native population of exosomal surface proteins that might ensure the desirable ligand–receptor interaction with the proposed target cell. Finding this optimal producer-target cell combination is vital to producing exosomes for therapeutic application. Notwithstanding these potential shortcomings and reservations, this area of research is highly dynamic and promises novel approaches to cancer patient diagnostics as well as therapy.
